# Gene Variants Associated With Individual Sensitivity for Taste Changes After the COVID‐19 Infection

**DOI:** 10.1155/bmri/5309217

**Published:** 2026-03-29

**Authors:** Lejla Pojskic, Ivona Kenjic, Belmina Saric Medic, Nikolina Tomic, Naris Pojskic, Jasmin Ramic, Naida Lojo Kadric

**Affiliations:** ^1^ Institute for Genetic Engineering and Biotechnology, University of Sarajevo, Sarajevo, Bosnia and Herzegovina, unsa.ba; ^2^ Department of Biology, Faculty of Science, University of Sarajevo, Sarajevo, Bosnia and Herzegovina, unsa.ba

**Keywords:** COVID-19, genetic propensity, postinfectious dysgeusia, smell and taste dysfunction, taste factors

## Abstract

Human gustatory function is a complex trait combining taste, smell, and touch required for the safety and quality assessment of ingested food. Taste dysfunction is one of the most prominent symptoms of COVID‐19 that was reversible in most cases, but some patients reported permanent changes in their perception of different food sources. This symptom brought attention to the complexity of the regulation of smell and taste and their potential use in diagnostics and treatment of acute and chronic taste disorders. We investigated the genetic association of candidate genes with SARS‐CoV‐2 infection–related dysgeusia. A total of 96 individuals with confirmed virus infection were divided into groups according to the presence of self‐reported taste dysfunction and genotyped using a custom Illumina gene panel. Out of 18 functionally related taste genes, statistically significant differences were observed for *HCN4* variants c∗2393C > G (*p* = 0.013) and c.2556G > A (*p* = 0.026), *PLCB2* variants c.3037‐55T > C (*p* = 0.019) and c.582+958_582+959inv (*p* = 0.021), and *TAS1R1* variant c.1594+41G > A (*p* = 0.03), which indicate possible association to taste dysfunction in response to virus infection.

**Summary**

• Genetic analysis revealed significant associations with five polymorphisms: HCN4 (c.∗2393C > G and c.2556G > A), PLCB2 (c.3037‐55T > C and c.582+958_582+959inv), and TAS1R1 (c.1594+41G > A).
• These findings suggest a potential genetic basis for taste perception changes after coronavirus disease 2019 (COVID‐19).
• Further studies with larger and more diverse populations are needed to validate these associations.

## 1. Introduction

Human gustatory function, encompassing taste, smell, and touch, is a physiological trait necessary for assessing food safety and quality, an area significantly advanced by recent research. Human gustatory experience is further supported with multiple and simultaneous interactions of smell, vision, and hearing that evoke a strong effect in brain areas responsible for taste perception [1]. One of the most prominent symptoms of COVID‐19 is a dysfunction of taste (dysgeusia) and smell (dysosmia) that was reversible in most cases, but some patients reported permanent changes in their perception of different food sources. In a large study of 12 European medical centers, up to 88% of patients with confirmed COVID‐19 reported olfactory and/or gustatory dysfunction (OGD) [2]. According to the study by Jensen et al. [3], most individuals regain normal function 30 days after first symptom occurrence, but approximately 20% report persistent OGD 6 months after infection. Other authors [4] propose significant changes in taste as a risk factor for cognitive decline in elderly COVID‐19 patients.

Major difficulties in studying COVID‐19‐related taste dysfunction stem from the challenges in objectively measuring the type, severity, and chronicity of symptoms, compounded by the variability in SARS‐CoV‐2 virulence. A deeper investigation into the genetic basis of infection‐initiated taste loss is necessary to explain its physiological effects. Taste receptors represent a connection between the internal and external environment, and their expression and function are genetically encoded. Taste receptors are chemoreceptors that interact with taste stimuli and initiate signals in our brains, allowing us to process different tastes. The G protein–coupled receptors (GPCRs) of the Taste Receptor Type 1 (T1R) family play a central role in the reception of sweet and umami taste in humans. The T1R family includes three genes: *TAS1R1*, *TAS1R2*, and *TAS1R3* [5]. *TAS1R1* and *TAS1R3* encode GPCRs, which together form a heterodimeric receptor capable of eliciting an umami taste response [6], while *TAS1R2* exclusively contributes to sweet taste receptors [7]. The T2R gene family governs the large human variation in bitter taste perception. This perceptual observation is primarily observed for specific bitter reception genes: *TAS2R38*, *TAS2R16*, and *TAS2R31* [8].

Existing studies suggest there are at least two transduction pathways for salty taste in humans: amiloride‐sensitive and amiloride‐insensitive, each possessing distinct properties [9–11]. The epithelial sodium channel (ENaC) has been proposed as a candidate component of the amiloride‐sensitive pathway [12]. The *TRPV1* gene encodes the receptor for capsaicin and other sharp chemical stimuli. TRPV1 receptors and their variants may provide a molecular mechanism explaining the unpleasant taste of sweeteners and salts [13]. The sour taste receptor is likely an ion channel, and several gene candidates have been proposed. The discovery of the *OTOP1* gene has advanced our understanding of sour taste, vestibular function, hydrogen ion (H^+^) transfer, and metalloprotein function [14], though a definite sour receptor remains unproven.

There are several genes that primarily have other functions in the body but are involved in taste perception either through cascading processes or by contributing to taste signal transduction pathways. As key multifunctional components of GPCR complexes, members of the ARRB2 family of proteins are involved in the desensitization of receptors and cause attenuation of cellular responses to stimuli. These proteins mediate a wide range of biological processes, including the perception of taste and smell [15]. Receptors TAS1R3 and TAS1R2 showed significant binding to calmodulin, while TAS1R1 showed a weaker binding affinity [16]. Cluster of differentiation 36 (*CD36*) encodes a protein that may have important functions as a cell adhesion molecule. Variants of the *CD36* gene are thought to mediate differences in the perception of dietary fat molecules [17]. The *GNAT3* gene encodes the alpha subunit of the heterotrimeric G protein, which is found not only in the oral epithelium but also in the tissues of the intestine. Research indicates an association between *GNAT3* polymorphisms and taste disorders [18].

The *HCN4* gene encodes a protein that is a member of cyclic nucleotide potassium channels activated by hyperpolarization. *HCN4* transcripts have been detected in taste buds [19], suggesting that this channel can also mediate responses to acidic stimuli. The *KCNJ2* gene encodes an integral membrane protein and a potassium channel. These channels are present in most mammalian cells and are involved in a wide range of physiological responses. *KCNJ2* polymorphisms are associated with sour taste variations and nontaste functions. *KCNJ2* is found in Acidity‐Sensing Type III taste cells and is associated with the extent of acidic taste transduction [20].

The *PLCB2* gene encodes a phosphodiesterase protein that catalyzes the hydrolysis of phosphatidylinositol 4,5‐bisphosphate to other transporters of inositol 1,4,5‐trisphosphate (IP3) and diacylglycerol. The encoded protein activates G proteins and is involved in the Type 2 flavor receptor signal transduction pathway. *PLCB2* transcripts are confirmed components of the taste signaling pathway [21].

Current evidence suggests that smell and taste disorders likely arise due to primary damage to olfactory sensory neurons and taste buds, mainly mediated by infection, inflammation, and subsequent dysfunction of supporting nonneuronal cells in the mucosa. However, the involvement of other mechanisms leading to chemosensory dysfunction has also been hypothesized [22].

Some research suggests that SARS‐CoV‐2 could spread from the respiratory system to the brain via receptors in sustentacular cells localized within the olfactory epithelium. The virus invades human cells via a binding receptor, ACE2 and TMPRSS2, facilitating viral penetration [23].

Taste receptor cells are highly susceptible to inflammatory processes, and evidence suggests that the cytokine storm associated with COVID‐19 can induce secondary damage to gustatory function due to the expression of ACE2 receptors within taste buds [24]. Furthermore, SARS‐CoV‐2 infection and the ensuing immune response have been shown to alter the morphology of taste buds, thereby contributing to taste dysfunction in affected individuals [25]. Recent investigations into postviral taste genetics have identified specific polymorphisms within TAS2R family genes that are associated with COVID‐19 susceptibility, as well as variations in antispike and antinucleocapsid antibody responses [26].

The objective of this study was to investigate the association between gene variants involved in taste physiology and symptoms of altered gustatory and/or olfactory function in SARS‐CoV‐2 infection. We also aimed to examine genetic polymorphisms in taste receptor genes and genes related to taste perception, as limited research is currently available on this topic. This knowledge could contribute to improving the diagnosis of virus‐related taste loss and potentially open new avenues for targeted and more effective treatment of individuals with taste and/or smell dysfunction.

## 2. Material and Methods

### 2.1. Ethics Statement and Subject Recruitment

The ethical aspects of the study′s objectives and methodology were approved by the Institutional Ethics Committee of the University of Sarajevo—Institute for Genetic Engineering and Biotechnology (Approval No. 552/21, dated November 29, 2021). The study included 100 unrelated individuals who provided written informed consent prior to the collection of biological samples and personal data. All participants had a confirmed COVID‐19 infection at least once during the pandemic period (2020–2022).

### 2.2. Sample Collection and Phenotyping

The study involved 100 unrelated, nonconsanguineous volunteers. All participants were nonclinical, healthy individuals who consented to provide information and biological specimens for the research purposes. All volunteers had a documented SARS‐CoV‐2 infection during the pandemic; the cohort was divided equally, with 50 individuals with OGD (OGD group) and 50 participants without dysfunction (no‐OGD group).

Inclusion criteria were as follows: age over 18 years, confirmed SARS‐CoV‐2 infection in the past year by a primary care physician after the PCR test, and no chronic diseases in personal medical history.

Exclusion criteria were as follows: age under 18 years, no prior SARS‐CoV‐2 infection, and the presence of chronic diseases.

Phenotypic data were collected via a 38‐question survey divided into three sections, which addressed personal and medical history regarding past SARS‐CoV‐2 infection and detailed food selectivity both before and after the onset of smell and/or taste disorders. The questionnaire was adapted based on MCSTQ‐Sc [27]. Questions were grouped into general health/demographics and the occurrence of smell and taste disorders.

The procedures for biological sample collection involved obtaining buccal swab samples for deoxyribonucleic acid (DNA) and RNA isolation by obtaining four swabs per person, rubbing the inner surface of both cheeks with a cotton swab. These were subsequently dried and transferred to a sterile tube for DNA extraction.

### 2.3. DNA Isolation and Quantification

Genomic DNA was isolated from buccal mucosa swab samples using a protocol for DNA isolation from peripheral blood [28], with a minor modification of the initial digestion step. Incubation was performed for 1 h at a 60°C thermoblock heater. DNA quantification was performed using QubitTM dsDNA HS Assay Kit (Invitrogen, Thermo Fisher Scientific, United States), according to the manufacturer′s instructions. DNA samples were diluted to an initial concentration of 10 ng/*μ*L prior to DNA sequencing preparations. All DNA was resolubilized in injection‐ready, DNAse‐ and RNAse‐free water and stored at −20°C.

### 2.4. Next‐Generation Sequencing (NGS)

Targeted gene sequencing was performed using the customized gene enrichment panel (Illumina, United States). The panel included functionally associated gustatory genes found in the literature, as well as several proinflammatory factors previously associated with loss of smell and taste. This panel also included several proinflammatory factors associated with loss of smell and taste. Of the genes in the original panel, 18 were selected for this research, categorized as “taste receptors” and “taste perception.” A list of these selected genes, their genomic location, function, and the literature references is listed in Table S1.

Library preparation for sequencing was conducted using Ampliseq Library Plus (Ref. 20019102, Illumina Inc., United States) and Ampliseq CD Set A (Ref. 20019105, Illumina Inc., United States), according to the manufacturer′s protocol. Once the quality and quantity of the template were confirmed, with the Qubit 2.0 method, the prepared libraries were diluted to a final concentration of 2 nM and pooled, then denatured, and further diluted to a final concentration of 1.5 pM before application to the NGS cartridge. The final library was loaded into the Illumina MiniSeq System (Illumina Inc., United States) using the MiniSeqTM Mid Output Reagent Cartridge 300 cycles (Ref. 20001311, Illumina Inc., United States) and the MiniSeq Flow Cell (Ref. 15073184, Illumina Inc., United States). Sequencing results were downloaded in (VCF format) and used for subsequent analysis.

### 2.5. Variant Annotation and Database Verification

All identified polymorphisms were cross‐checked in publicly available genomic databases. Population allele frequencies were obtained from gnomAD [29], clinical significance and pathogenicity classifications were retrieved from ClinVar [30], and reference SNP identifiers (rsIDs) were confirmed using dbSNP [31].

### 2.6. Statistical Analysis

Biostatistical analysis was performed to test the association of the observed gene variants with the phenotypic parameters. The Chi‐square (*X* ^2^) test and Fisher′s exact test were used to examine the significance of differences in allele frequency distribution between the two study groups (OGD group vs. no‐OGD group).

To control for multiple comparisons, the Benjamini–Hochberg (BH) procedure was applied at a false discovery rate (FDR) of 0.05 to adjust *p* values and identify statistically significant associations while limiting the expected proportion of false positives.

## 3. Results

### 3.1. Phenotyping Results

The study cohort comprised 100 participants (50 males and 50 females) with a previously confirmed SARS CoV‐2 infection. Four samples were excluded from the final analysis due to low genotyping quality following NGS, resulting in a total sample size of 96.

Demographic characteristics (age, sex, smoking status, and history of head injury) were assessed for all participants (Table 1). There were no statistically significant differences in these baseline parameters between the case group (OGD) and the control group (no‐OGD), indicating successful matching.

**Table 1:** Demographic comparison table between case and control groups.

Demographic parameter	OGD group	No‐OGD group	Total cohort
Sex	48	48	96
Smoking status—active	28	25	53
Smoking status—passive	38	37	75
Head injury	2	0	2

**Figure 1:**
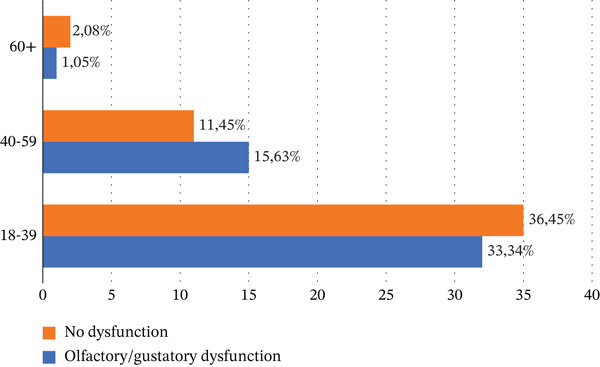
Age distribution of participants in this study per group.

Age distribution was stratified into three brackets: 18–39, 39–59, and 60+ years. The results of this age distribution across the entire cohort are presented in Figure 1. As shown in Figure 1, the largest proportion of participants fell into the 18–39 age bracket (69.79% of the total cohort), providing balanced representation across adult age groups.

**Figure 2:**
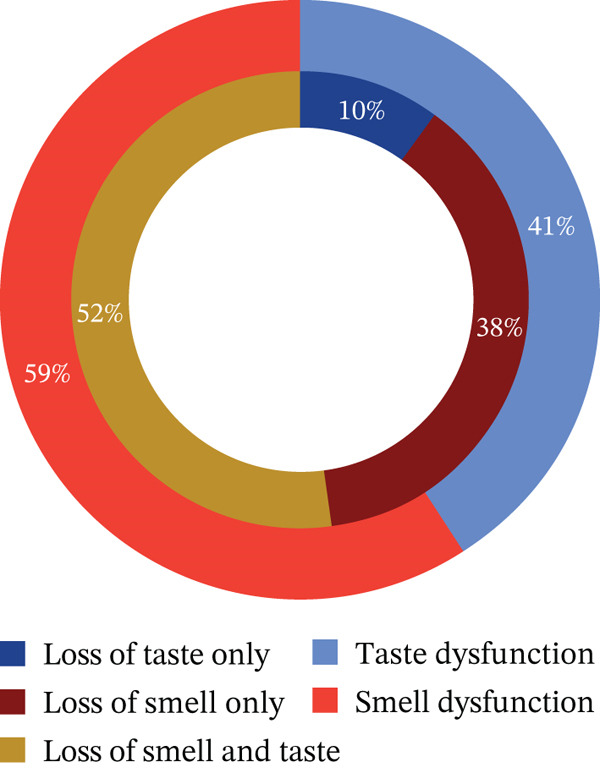
Distribution of olfactory and gustatory dysfunction among participants, highlighting specific loss patterns.

Based on self‐reported responses to the questionnaire, the distribution of participants reporting loss of taste or smell with a specific loss pattern is illustrated in Figure 2. The figure, a nested donut plot, details the specific loss patterns: The outer layer represents the overall percentage of participants experiencing OGD, while the inner layer shows the proportion of subjects with loss of smell only, loss of taste only, or loss of both senses.

### 3.2. NGS Results

Genetic analysis was performed on 96 participants; four samples were excluded due to low sequencing quality. The custom panel targeted 18 genes highly specific to taste perception (Table S1).

**Figure 3:**
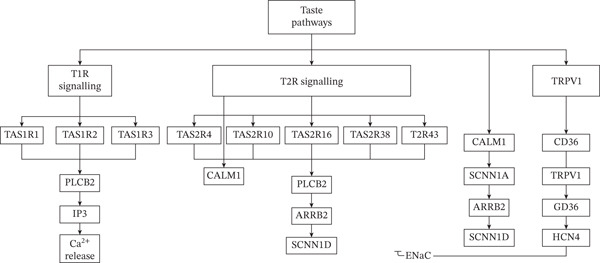
Taste signalling pathways: integration of T1R, T2R, ENaC, and TRPV1 mechanisms.

Gene selection for the custom panel was based on strong biological plausibility and pathway enrichment. The inclusion criteria prioritized genes with direct functional involvement in the primary gustatory signal transduction pathway. Specifically, this included genes encoding the primary taste receptors for the five basic tastes (e.g., TAS1R and TAS2R families), as well as genes encoding ion channels critical for salt or sour transduction, or general membrane potential regulation in taste cells (e.g., SCNN1AD, KCNJ2, and HCN4). Furthermore, the panel incorporated key signal transduction components necessary for receptor function and regulation of the entire signaling cascade downstream of the T1R and T2R receptors (e.g., PLCB2 and GNAT3). Panel genes are illustrated in Figure 3, which presents the major molecular components and signaling cascades involved in taste perception. Sweet and umami stimuli activate T1R receptors (TAS1R family) via GNAT3 and PLCB2, leading to IP_3_‐mediated Ca^2+^ release. Bitter compounds engage T2R receptors (TAS2R family), triggering similar PLCB2/IP_3_/Ca^2+^ signaling and downstream modulators such as CALM1 and ARRB2. Salty taste is mediated by ENaC channels (SCNN1A and SCNN1D), while fat and pungent stimuli involve TRPV1 and CD36, integrating with ion channels (HCN4) and arrestin pathways. Shared components like CALM1 and GNAT3 highlight cross‐talk between taste modalities. A complete list of these 18 genes, including their genomic location, function, and supporting literature references used during panel design, is provided in Table S1.

The results of the gene association analysis for 18 taste‐related genes and their variants are provided in Table S2. Comprehensive sequencing of these genes revealed more than 300 distinct genetic variants across the study cohort. These variants exhibited varying distribution patterns: Some were recurrent and detected in multiple samples, whereas others were unique to individual samples. The majority of identified variants were missense mutations, which are known to potentially alter protein function, followed by structural changes such as deletions and duplications. These findings underscore the genetic diversity within taste‐related genes and suggest possible functional implications for taste perception. Table S2 provides a complete overview of all detected variants. Statistically significant findings are presented in Table 2.

**Table 2:** Fisher′s exact value, *p* values, and adjusted *p* values for tested variants under BH‐FDR.

Gene	Variant	Fisher′s exact value	*p* value	BH adjusted *p* value
*HCN4*	c.∗2393C > G	6337	0.013	0.030
	c.2556G > A	5188	0.026	0.030
*PLCB2*	c.3037‐55T > C	5224	0.019	0.030
	c.582+958_582+959inv	7692	0.021	0.030
*TAS1R1*	c.1594+41G > A	4471	0.03	0.030

A positive genetic association was observed for two polymorphisms of *HCN4* (c.∗2393C > G and c.2556G > A), two polymorphisms of *PLCB2* (c.3037‐55T > C and c.582+958_582+959inv), and one *TAS1R1* polymorphism (c.1594+41G > A). Raw *p* values ranged from 0.013 to 0.030 (Table 2). To correct for multiple comparisons, we applied the BH procedure at a FDR of 0.05. After BH adjustment, all variants had adjusted *p* values of 0.030 and were considered significant at *q* ≤ 0.05. Allele frequencies for these five variants in *PLCB2*, *TAS1R1*, and *HCN4* genes were compared between participants with OGD and those without (no‐OGD) (Table 3). Odds ratios (ORs) ranged from 0.36 to 8.64, with PLCB2 c.3037‐55T > C showing a protective association (OR = 0.39; CI: 0.21–0.73), while *HCN4* variants exhibited strong risk associations (OR > 7). Hardy–Weinberg equilibrium (HWE) was maintained for most variants, except *PLCB2* c.3037‐55T > C, which showed marked deviation in both groups.

**Table 3:** Allele frequencies with odds ratios for variants in the OGD vs. no‐OGD groups.

Gene	*PLCB2*		*TAS1R1*	*HCN4*	
Variant	c.3037‐55T > C	c.582+958_582+959inv	c.1594+41G > A	c.∗2393C > G	c.2556G > A
*p* (A) OGD	0.25	0.625	0.8438	0.9896	0.9896
*q* (a) OGD	0.75	0.375	0.1562	0.0104	0.0104
*p* (A) no‐OGD	0.4583	0.5417	0.9375	0.9167	0.9271
*q* (a) no‐OGD	0.5417	0.4583	0.0625	0.0833	0.0729
*p* (A) total	0.3542	0.5833	0.8906	0.9531	0.9583
*q* (a) total	0.6458	0.4167	0.1094	0.0469	0.0417
OR (A)	0.3939	1.4103	0.36	8.6364	7.4719
95% CI	(0.21, 0.73)	(0.79, 2.51)	(0.13, 0.97)	(1.06, 70.46)	(0.90, 61.95)

## 4. Discussion

Our findings in this study suggest that ageusia (loss of taste) is a significant symptom experienced by a proportion of people after SARS‐CoV‐2 infection. The 15% incidence observed here may appear relatively low when compared to some published studies. In a study carried out in 2020, 29.7% of respondents reported a loss of sense of taste [32], while a review analysis, carried out in the same year, encompassing 16 selected studies, found that the incidence of smell and taste dysfunction in Europe ranged from 34% to 86% [33]. Nonetheless, the percentage obtained in this study could be clinically significant because ageusia can affect quality of life and nutrition and potentially contribute to prolonged recovery in some individuals.

Although loss of taste is one of the symptoms of COVID‐19, the link between genetics and loss of taste has not been sufficiently investigated. There are certain genetic conditions that can affect the sense of smell and taste, but it remains unclear whether these directly influence taste loss following SARS‐CoV‐2 infection. Several studies have investigated the genetic basis of loss of taste (and smell) after COVID‐19. In one of them, two genes, UDP Glucuronosyltransferase Family 2 Member A1 (*UGT2A1*) and UDP Glucuronosyltransferase Family 2 Member A2 (*UGT2A2*), were found to be associated with these symptoms. [34]. Our study did not find a statistically significant association for the *UGT2A1* and *UGT2A2* variants (or other variants reported in the literature) with OGD following SARS‐CoV‐2 infection. This discrepancy with previous findings, such as those reported by Shelton et al. [34], can be attributed to several critical differences in methodology and subject demographics. Therefore, the absence of a significant association for the previously reported *UGT2A* variants in our cohort should be interpreted primarily because of the limited statistical power inherent to our sample size, rather than evidence against the biological validity of the published findings. Future studies with larger, prospectively collected cohorts in this region would be necessary to validate these specific genetic links.

Results of this study suggest a possible association between certain gene variants and loss of taste after SARS‐CoV‐2 infection, which may be intriguing. This would potentially mean that certain variants of the *HCN4* gene affect the function of neurons in the nose and mouth that are affected by the virus, variants in the *PLCB2* genes modify the response to viral infection in a way that leads to a loss of the sense of taste, and *TAS1R1* variants affect the function or expressiveness of this receptor, which may contribute to changes in taste perception after infection.

Regarding the findings within *HCN4* gene polymorphism, specific information about the phenotypic effects of the c.∗2393C > A variant has not been documented, but changes in the 3 ^′^ untranslated region (UTR) can affect messenger ribonucleic acid (mRNA) stability, which may further affect gene expression and protein functionality, while the c.2556G > A variant results in nucleotide substitution within the coding sequence of the *HCN4* gene, which can cause an amino acid change in the protein product. HCN4 c.∗2393C > G (rs74933243) shows a global minor allele frequency ~4.9% in gnomAD with ClinVar benign classification—consistent with a noncoding 3 ^′^ UTR variant less likely to disrupt protein sequence (VCV000885567). *HCN4* c.2556G > A (p.Pro852=), rs117819825, is a synonymous change with benign submissions from multiple laboratories and appears to be rare (MAF typically < 1%), again tempering expectations of a large functional impact in the absence of splicing or regulatory effects (RCV000081298). However, there is no prior functional evidence that the c.∗2393C > A and c.2556G > A variants in the *HCN4* gene are associated with taste loss.

The *PLCB2* gene variants identified in this study are located in noncoding regions. Specifically, the c.3037‐55T > C variant is found in an intron and, although not directly translated into the protein, could potentially affect the splicing process. The c.582+958_582+959inv variant indicates an inversion within introns 6–7, which may alter the functionality of the resulting protein. While the *PLCB2* gene is well established for its role in taste signal transduction, the direct link between these specific variants and the loss of the sense of taste has not been widely reported in currently available scientific data. However, mutations and variations in genes involved in taste signaling, such as *PLCB2*, could affect taste perception. The *PLCB2* c.582+958_582+959inv (rs8007267) variant is common globally (minor allele frequency approximately 0.21), with noticeable population variability (e.g., higher frequency in African ancestries at approximately 0.59 and lower in Europeans at approximately 0.18). This breadth of variability suggests that any observed case–control association could be confounded by population substructure or ancestry unless appropriately controlled. The *PLCB2* c.3037‐55T > C variant demonstrated a striking deviation from HWE (Chi‐square approximately 48, *p* < 0.0001) in both the OGD (case) and no‐OGD (control) groups. This disequilibrium was driven by the complete absence of heterozygotes despite the allele frequencies predicting their presence—an observation that strongly suggests potential technical issues (e.g., genotyping error, allele dropout, or miscalls). Alternatively, the deviation could reflect a strong population substructure or, in rarer scenarios, biological selection against heterozygotes. Given the strength of the HWE disequilibrium and the protective association observed for this variant (OR approximately 0.39, 95% confidence interval: 0.21–0.73), further validation using independent cohorts is warranted before inferring a causal relationship.

The c.1594+41G > A variant is in the intron region of the *TAS1R1* gene. If the variant were to cause the protein to fold incorrectly, it could affect the receptor′s ability to recognize umami, which could manifest as changes in the perception of this taste. To date, there is research that polymorphisms of the *TAS1R1* gene contribute to differences in the intensity of human taste, but specific studies directly linking the c.1594+41G > A variant to loss of the sense of taste are not currently available. However, there is evidence that variations in the genes that code for taste receptors can affect the perception of taste. Finally, *TAS1R1* c.1594+41G > A do not appear in dbSNP/gnomAD (to our search), suggesting novel or extremely rare status; this underscores the need for replication and functional assessment before drawing firm conclusions (contrast shown by *TAS1R1* entries such as rs74049682 at different positions).

The application of multiple comparison corrections substantially influenced the interpretation of our results. Using BH‐FDR at *q* = 0.05, all five variants were retained as significant, suggesting potential associations that merit further investigation. Given the exploratory nature of this analysis and the biological plausibility of these variants in taste signaling pathways, the FDR‐based findings provide a reasonable basis for prioritizing these loci for replication in larger cohorts. Our results emphasize the importance of considering allele frequency and effect size in study design and interpretation. Future studies with larger sample sizes or meta‐analyses are needed to validate these associations.

The interpretation of the identified genetic associations, particularly the protective effect of the *PLCB2* variant, should be considered within the context of potential linkage disequilibrium (LD) and gene–environment interactions. It is possible that the variants analyzed in our custom panel are in LD with other functional polymorphisms or regulatory elements located in nearby genomic regions that were not sequenced. These linked variants could independently or synergistically influence the taste‐signaling phenotype. Furthermore, the manifestation of ageusia following SARS‐CoV‐2 infection likely involves a complex interaction mediated by the host′s inflammatory response. Proinflammatory cytokines, such as Interleukin‐6 (IL‐6) and tumor necrosis factor‐alpha (TNF‐*α*), are frequently elevated during the acute phase of COVID‐19 and have been shown to correlate directly with the severity of taste and smell disorders [35]. These cytokines can acutely modulate taste bud function and inhibit the renewal of taste bud progenitor cells [36]. Genetic variants in signal transduction components, such as *PLCB2*, may alter the threshold at which these inflammatory stimuli disrupt signaling. Specifically, TNF‐*α* has been shown to be expressed in Type II taste cells, where it can interfere with the transduction pathways necessary for bitter, sweet, and umami perception [37]. The resulting “cytokine storm” may therefore trigger apoptosis in taste buds or disrupt the regeneration of the taste epithelium [38]. Consequently, the observed genetic susceptibility may be contingent upon the individual′s systemic or localized inflammatory profile.

The reliance on self‐reported gustatory dysfunction, collected using the MCSTQ‐Sc questionnaire, introduces a potential bias because it is a subjective rather than an objective parameter. Most of the prior research into COVID‐19‐related dysgeusia has similarly utilized subjective parameters [39–41]. Lacking objective measures for taste dysfunction in this study, we were required to rely on self‐reporting. This subjective limitation may potentially weaken the strength of the observed genotype–phenotype associations.

As a conclusion, there are no studies in the current literature that confirm the link between specific genes *TAS1R1*, *HCN4*, and *PLCB2* and their variants, with the loss of the sense of taste. Although these genes are involved in various biological functions, available data do not indicate a direct relationship between these gene variants and loss of the sense of taste, which reflects the importance of pursuing this direction of research and can impede diagnostics. As the diagnostic limitation of this method and polymorphisms, it is important to note that further research results should confirm these links and suggest the mechanisms through which specific gene variants can affect the taste dysfunction. These results may help better understand individual variability in the symptoms and consequences of viral infections, including SARS‐CoV‐2.

## 5. Conclusions

The results of the analysis showed significant differences in the frequency of certain allelic variants between these two groups. Statistically significant differences were observed for three of the 18 analyzed genes, namely, *HCN4* with variants c.∗2393C > G and c.2556G > A, *PLCB2* with variants c.3037‐55T > C and c.582+958_582+959inv, and *TAS1R1* with variant c.1594+41G > A, which indicates a potential genetic predisposition to this symptom and suggests a possible mechanism through which the virus affects the nervous system and sensory functions. This is one of the first exploratory findings suggesting potential associations of “taste” genes with dysgeusia and/or dysosmia after SARS‐CoV‐2 infection and is important for further research into the unknown consequences of infection and their prevention or inflammatory treatment. The results provide a basis for future research and may have significant implications for personalized medicine in the context of post‐COVID‐19 syndrome, especially regarding the prevention and treatment of sensory loss.

## Author Contributions

**Lejla Pojskic:** conceptualization, methodology, supervision, writing – original draft. **Ivona Kenjic:** data curation, visualization, writing – original draft. **Belmina Saric Medic:** investigation, data curation, formal analysis, writing – original draft. **Nikolina Tomic:** investigation, data curation, formal analysis, writing – original draft. **Naris Pojskic:** formal analysis, supervision, writing – review and editing. **Jasmin Ramic:** investigation, data curation, writing – review and editing. **Naida Lojo Kadric:** conceptualization, funding acquisition, project administration, data curation, formal analysis, writing – review and editing.

## Funding

This work is supported by the grant of the Ministry of Science, Higher Education and Youth of Sarajevo Canton (contract no.: 27‐02‐11‐41250‐40/21).

## Disclosure

All authors have reviewed and approved the manuscript and agree to be fully accountable for the integrity and accuracy of the work. We did not have contributors who were not authors. We understand and accept that no further changes to authorship will be possible.

## Conflicts of Interest

The authors declare no conflicts of interest.

## Supporting Information

Additional supporting information can be found online in the Supporting Information section.

## Supporting information


**Supporting Information 1** Table S1: List of “taste” related genes and their function in the customized panel used for NGS.

**Supporting Information 2** Table S2: Genetic association results for all identified variants (statistically significant findings are marked in bold).

## Data Availability

The data that supports the findings of this study are available in the supporting information of this article.
