# Supplementary material for: Gene Variants Associated With Individual Sensitivity for Taste Changes After the COVID‐19 Infection
Source: Biomed Res Int. 2026 Mar 29;2026:5309217. doi: 10.1155/bmri/5309217 (PMC13140813; doi:10.1155/bmri/5309217)
Supplement: Supplementary file 1 — Supporting Information 1 Table S1: List of “taste” related genes and their function in the customized panel used for NGS. [file BMRI-2026-5309217-s002.docx]

*Supplementary Table 1.* – List of “taste” related genes and their function in customized panel used for NGS sequencing

| Gene | Locus | Function | Literature reference |
| --- | --- | --- | --- |
| ARRB2 | chr17:4,710,596-4,721,500 | G-protein adenylyl cyclase (AC), cyclic nucleotide (CNG) ion channel, and several other proteins, calmodulin (CALM), phosphodiesterase (PDE), ß-arrestin2 (ARRB2), some kinases (PKA,GRK3,ORK), and RGS2 protein (regulator of G-protein signaling) participate in the feedback mechanisms that olfactory and gustatory sensory neurons use to adjust their sensitivity | Canals et al. (2019) |
| CALM1 | chr14:90,396,502-90,408,268 |  |  |
| CD36 | chr7:80,369,575-80,679,277 | Variants of *the CD36 gene*  are responsible for differences in the ability to perceive fat molecules contained in food. Many variants of *CD36* have a consequential impact on body mass index (BMI) and risk of developing obesity | Abdoul-Azize et al. (2014) |
| GNAT3 | chr7:80,458,635-80,512,064 | Signal Transduction for Flavors | Hwang et al. (2019) |
| HCN4 | chr15:73,319,859-73,368,958 | Gene transcripts have been detected in taste buds and proposed as candidates for acidic taste receptors | Gao et al. (2009) |
| KCNJ2 | chr17:70,168,673-70,180,044 | Gene product is found in acidity-sensing type III taste cells and is associated with the extent of acidic taste transduction | Ferraris et al. (2021) |
| PLCB2 | chr15:40,278,176-40,307,945 | Signal Transduction for Flavors | Sukumaran et al. (2017) |
| SCNN1A | chr12:6,346,843-6,377,730 | ENaC family - salty receptors (associated with high salt sensitivity; risk of hypertension) | Dias et al. (2013) |
| SCNN1D | chr1:1,280,436-1,292,029 |  |  |
| TAS1R1 | chr1:6,555,307-6,579,755 | Sweet Taste Perception Potential Relationship with  Individual  Differences  in umami sensitivity | Shigemura et al. (2009) |
| TAS1R2 | chr1:18,839,599-18,859,682 |  |  |
| TAS1R3 | chr1:1,331,280-1,335,320 |  |  |
| TAS2R4 | chr12:11,091,287-11,092,313 | Bitter taste receptors (for denatonium) | Pulkkinen et al. (2012) |
| TAS2R10 | chr12:10,825,317-10,826,358 |  |  |
| TAS2R16 | chr7:122,994,704-122,995,700 | Bitter taste receptor activated by bitter β-glucopyranosides | Bufe et al. (2002) |
| TAS2R38 | chr7:141,972,631-141,973,773 | Associated with high sensitivity to the bitter taste of phenylthiocarbamide (PTC) compounds and similar molecules in food or beverages and is the cause of metabolic diseases | Robino et al. (2021) |
| TAS2R43 | chr7:141,776,674-141,781,691 | Determines increased sensitivity to saccharin bitterness | Sandau et al. (2015) |
| TRPV1 | chr17:3,565,444-3,609,411 | Vanillin1 -receptor for salt; TRPV1 can indirectly affect some primary taste qualities through the release of substance P and a peptide associated with the calcitonin gene. | Rhyu et al. (2021) |
