# Supplementary material for: Gene Variants Associated With Individual Sensitivity for Taste Changes After the COVID‐19 Infection
Source: Biomed Res Int. 2026 Mar 29;2026:5309217. doi: 10.1155/bmri/5309217 (PMC13140813; doi:10.1155/bmri/5309217)
Supplement: Supplementary file 2 — Supporting Information 2 Table S2: Genetic association results for all identified variants (statistically significant findings are marked in bold). [file BMRI-2026-5309217-s001.docx]

*Supplementary Table 2* – Genetic association results for all identified variants (statistically significant findings are marked bold).

| *Gene* | *Identified variants* | *Fisher Exact test* | *P value* |
| --- | --- | --- | --- |
| *ARBB2* | c.424G>A | 0,969 | 0,51 |
|  | c.1208C>T | 1,054 | 0,49 |
|  | c.357C>A | 0,969 | 0,51 |
|  | c.421-14C>T | 2,628 | 0,269 |
|  | c.480+65C>G | 0,969 | 0,51 |
|  | c.481-52T>C | 1,157 | 0,561 |
|  | c.481-83A>G | 1,157 | 0,561 |
|  | c.548+26_549-58del | 0,389 | 0,484 |
|  | c.55-82G>A | 0,969 | 0,51 |
|  | c.639C>T | 0,001 | 0,742 |
|  | c.843-27A>G | 3,354 | 0,187 |
|  | c.843-62T>G | 0,969 | 0,51 |
|  | c.903C>T | 0,523 | 0,77 |
|  | c.927C>T | 1,959 | 0,258 |
|  | c.981-136C>T | 3,248 | 0,197 |
|  | c.981-55G>A | 3,248 | 0,197 |
| *CALM1* | c.*1070C>G | 0,969 | 0,51 |
|  | c.*1082A>G | 0,003 | 0,641 |
|  | c.*1183_*1184del | 0,959 | 0,324 |
|  | c.*1184del | 0,358 | 0,836 |
|  | c.*1304T>A | 0,01 | 0,742 |
|  | c.*1430T>C | 0,969 | 0,51 |
|  | c.*1439_*1441del | 1,054 | 0,49 |
|  | c.*1440-*1441del | 1,541 | 0,171 |
|  | c.*1441del | 2,657 | 0,78 |
|  | c.*1444C>G | 2,809 | 0,087 |
|  | c.*1518G>C | 0,969 | 0,51 |
|  | c.*1952T>G | 0,545 | 0,344 |
|  | c.*2085A>G | 1,054 | 0,49 |
|  | c.*2250_*2251delinsGC | 0,01 | 0,742 |
|  | c.*2351C>G | 1,822 | 0,402 |
|  | c.*2737C>T | 1,226 | 0,542 |
|  | c.*2861_*2864del | 0,389 | 0,484 |
|  | c.*2943T>C | 0,969 | 0,51 |
|  | c.*3218G>A | 0,001 | 0,742 |
|  | c.*322G>T | 0,969 | 0,51 |
|  | c.*417T>C | 0,005 | 0,603 |
|  | c.*867_*868del | 1,959 | 0,258 |
|  | c.*868del | 0,002 | 0,676 |
|  | c.*868dup | 1,015 | 0,211 |
|  | c.179-33A>G | 1,052 | 0,49 |
|  | c.-218C>T | 1,866 | 0,393 |
|  | c.3+200G>A | 1,959 | 0,258 |
|  | c.-37_-36dup | 1,054 | 0,49 |
|  | c.-86G>A | 1,634 | 0,442 |
| *CD36* | c.*238_*253del | 2,066 | 0,356 |
|  | c.1126-150C>A | 3,335 | 0,189 |
|  | c.1126-178G>A | 1,054 | 0,49 |
|  | c.1199+52C>G | 1,054 | 0,49 |
|  | c.1199+58_1199+59inv | 1,054 | 0,49 |
|  | c.1199+64_199+65insGATACTACGATACTACAGAT | 1,054 | 0,49 |
|  | c.121-6T>C | 2,276 | 0,32 |
|  | c.-132A>C | 0,659 | 0,719 |
|  | c.133G>C | 1,054 | 0,49 |
|  | c.-183-32dup | 0,803 | 0,669 |
|  | c.-183-7449C>A | 0,894 | 0,639 |
|  | c.-183-7497G>C | 1.054 | 0,49 |
|  | c.-183-7500G>T | 1,265 | 0,531 |
|  | c.-183-7515C>A | 1,959 | 0,258 |
|  | c.-183-7573del | 0,128 | 0,459 |
|  | c.282-10A>G | 0,537 | 0,764 |
|  | c.286C>T | 1,054 | 0,49 |
|  | c.-375G>C | 5,059 | 0,08 |
|  | c.544T>C | 1,054 | 0,49 |
|  | c.573G>A | 0,169 | 0,52 |
|  | c.702-58T>C | 0,969 | 0,51 |
|  | c.702G>A | 0,969 | 0,51 |
|  | c.787_808del | 1,054 | 0,49 |
|  | c.879T>C | 0,001 | 0,742 |
|  | c.-89-227_-89-225del | 0,969 | 0,51 |
| *GNAT3* | c.1011C>T | 2,961 | 0,228 |
|  | c.1014A>G | 1,054 | 0,49 |
|  | c.118+56T>C | 1,003 | 0,606 |
|  | c.119-36A>G | 2,197 | 0,333 |
|  | c.119-48T>C | 0,001 | 0,742 |
|  | c.119-89dup | 2,197 | 0,333 |
|  | c.161+5G>T | 1,054 | 0,49 |
|  | c.161+71A>T | 0,64 | 0,334 |
|  | c.162-12T>C | 1,076 | 0,584 |
|  | c.461+41T>G | 0,969 | 0,51 |
|  | c.590+34A>G | 1,054 | 0,49 |
|  | c.591-34C>T | 1,664 | 0,435 |
|  | c.875-135A>G | 0,258 | 0,879 |
|  | c.875-179C>T | 2,129 | 0,237 |
|  | c.91G>A | 0,969 | 0,51 |
| *HCN4* | c.*1140G>A | 0,389 | 0,484 |
|  | c.*118_*119del | 0,038 | 0,981 |
|  | c.*1197T>C | 0,081 | 0,527 |
|  | c.*127A>G | 1,054 | 0,49 |
|  | c.*1490A>C | 0,169 | 0,52 |
|  | c.*1989G>A | 0,964 | 0,618 |
|  | c.*2354A>G | 2,97 | 0,129 |
|  | **c.*2393C>G** | **6,337** | **0,013** |
|  | c.*2445C>T | 2,671 | 0,112 |
|  | c.*383C>T | 0,969 | 0,51 |
|  | c.*414C>A | 0,007 | 0,996 |
|  | c.*702del | 0,035 | 0,983 |
|  | c.*708A>G | 1,226 | 0,542 |
|  | c.107G>A | 1,231 | 0,54 |
|  | c.1209+64G>T | 1,959 | 0,258 |
|  | c.-121C>T | 0,969 | 0,51 |
|  | c.-132G>C | 0,969 | 0,51 |
|  | c.1372-29A>G | 1,959 | 0,258 |
|  | c.1518C>T | 0,969 | 0,51 |
|  | c.1558C>T | 0,363 | 0,359 |
|  | c.1590+59C>T | 1,054 | 0,49 |
|  | c.1797G>A | 1,054 | 0,49 |
|  | c.1978+46G>A | 2,97 | 0,129 |
|  | c.1979-150G>A | 1,469 | 0,48 |
|  | c.1979-41A>G | 2,342 | 0,31 |
|  | **c.2556G>A** | **5,188** | **0,026** |
|  | c.2621T>C | 1,054 | 0,49 |
|  | c.-277_-268del | 0,303 | 0,516 |
|  | c.2917G>A | 2,129 | 0,237 |
|  | c.3337A>G | 1,133 | 0,293 |
|  | c.3600A>G | 1,226 | 0,542 |
|  | c.458A>G | 0,969 | 0,51 |
|  | c.-585C>A | 0,004 | 0,619 |
|  | c.-620C>G | 0,969 | 0,51 |
|  | c.785+32C>T | 0,969 | 0,51 |
| *KCNJ2* | c.*1069C>T | 1,388 | 0,5 |
|  | c.*1267A>G | 1,018 | 0,601 |
|  | c.*1596A>C | 1,054 | 0,49 |
|  | c.*1669T>A | 1,054 | 0,49 |
|  | c.*1678del | 2,863 | 0,075 |
|  | c.*1720_*1721del | 1,054 | 0,49 |
|  | c.*1721del | 2,258 | 0,101 |
|  | c.*1721dup | 2,368 | 0,121 |
|  | c.*1777G>T | 0,126 | 0,457 |
|  | c.*211T>C | 0,969 | 0,51 |
|  | c.*2262C>T | 0,001 | 0,742 |
|  | c.*2398A>G | 0,969 | 0,51 |
|  | c.*259_254del | 0,969 | 0,51 |
|  | c.*2508G>A | 0,969 | 0,51 |
|  | c.*3163T>A | 1,054 | 0,49 |
|  | c.*3463del | 1,071 | 0,238 |
|  | c.*79C>T | 2,129 | 0,237 |
|  | c.*917T>G | 0,969 | 0,51 |
|  | c.1146C>T | 1,069 | 0,586 |
|  | c.417C>A | 0,969 | 0,51 |
| *PLCB2* | c.*262T>C | 0,64 | 0,334 |
|  | c.*468C>T | 0,389 | 0,484 |
|  | c.*5C>A | 1,054 | 0,49 |
|  | c.1155+51T>G | 1,054 | 0,49 |
|  | c.1156-105G>C | 2,34 | 0,31 |
|  | c.1156-71G>A | 2,97 | 0,129 |
|  | c.1182C>T | 0,451 | 0,798 |
|  | c,1239-18C>T | 1,526 | 0,201 |
|  | c.1239-24C>T | 3,14 | 0,202 |
|  | c.1303G>A | 0,969 | 0,51 |
|  | c.1324-109G>C | 3,354 | 0,187 |
|  | c.1412C>T | 1,054 | 0,49 |
|  | c.-141T>A | 0,969 | 0,51 |
|  | c.1431T>C | 1,002 | 0,606 |
|  | c.163-8G>A | 0,799 | 0,671 |
|  | c.1782-61G>T | 0,969 | 0,51 |
|  | c.1907-114C>T | 0,969 | 0,51 |
|  | c.1907-54C>T | 0,969 | 0,51 |
|  | c.2227-55del | 0,969 | 0,51 |
|  | c.231+15T>G | 3,746 | 0,053 |
|  | c.231+75C>G | 1,746 | 0,418 |
|  | c.232-35C>T | 0,389 | 0,484 |
|  | c.2527-39G>T | 4,227 | 0,121 |
|  | c.2603-25A>C | 1,133 | 0,293 |
|  | c.2603-41C>T | 0,969 | 0,51 |
|  | c.2603-86C>A | 0,969 | 0,51 |
|  | c.2776C>A | 1,535 | 0,464 |
|  | c.2870+8T>C | 2,129 | 0,237 |
|  | **c.3037-55T>C** | **5,224** | **0,019** |
|  | c.3037-69A>C | 1,031 | 0,597 |
|  | c.3209+63_3209+64del | 3,481 | 0,175 |
|  | c.3267+51_3267+56del | 2,129 | 0,237 |
|  | c.3267+53_3267+56del | 2,427 | 0,297 |
|  | c.3267+55A>T | 3,229 | 0,113 |
|  | c.3267+57_3267+59delinsACA | 3,922 | 0,141 |
|  | c.3267+57T>A | 1,1 | 0,577 |
|  | c.3267+59_3267+61delinsACA | 4,577 | 0,101 |
|  | c.3267+59T>A | 0,001 | 0,742 |
|  | c.3267+61_3267+63delinsACA | 4,061 | 0,131 |
|  | c.3267+61T>A | 0,969 | 0,51 |
|  | c.3267+63_3267+65delinsACA | 1,959 | 0,258 |
|  | c.3267+63T>A | 0,969 | 0,51 |
|  | c.3267+65_3267+67delinsACA | 0,969 | 0,51 |
|  | c.3267+65T>A | 0,969 | 0,51 |
|  | c.3267+86del | 4,146 | 0,126 |
|  | c.3284A>G | 2,002 | 0,368 |
|  | c.3355-45G>T | 0,892 | 0,64 |
|  | c.3344-73C>T | 1,959 | 0,375 |
|  | c.537G>A | 0,969 | 0,51 |
|  | c.582+1176G>C | 2,002 | 0,368 |
|  | c.582+1183dup | 3,632 | 0,062 |
|  | c.582+141C>T | 1,054 | 0,49 |
|  | c.582*727C>T | 1,256 | 0,534 |
|  | **c.582+958_582+959inv** | **7,692** | **0,021** |
|  | c.582+958T>C | 0,001 | 0,742 |
|  | c.683+74C>T | 0,64 | 0,334 |
| *SCNN1A* | c.*296C>T | 0,969 | 0,51 |
|  | c.*633G>T | 0,389 | 0,484 |
|  | c.*850C>T | 1,054 | 0,49 |
|  | c.*914A>G | 1,133 | 0,293 |
|  | c.1052+146_1052+148delinsTGT | 0,969 | 0,51 |
|  | c.1052+146G>T | 0,069 | 0,966 |
|  | c.1155C>T | 1,054 | 0,49 |
|  | c.1177G>A | 0,257 | 0,48 |
|  | c.1419+19C>T | 1,054 | 0,49 |
|  | c.1419+54C>T | 0,998 | 0,607 |
|  | c.1537+47C>T | 1,054 | 0,49 |
|  | c.1654T>C | 1,246 | 0,223 |
|  | c.1674+56C>G | 0,258 | 0,879 |
|  | c.1730+22G>A | 1,054 | 0,49 |
|  | c.1730+32G>A | 0,139 | 0,933 |
|  | c.1806+57G>T | 0,969 | 0,51 |
|  | c.1806+63G>A | 0,969 | 0,51 |
|  | c.2164A>G | 3,059 | 0,217 |
|  | c.303G>A | 1,054 | 0,49 |
|  | c.593+71A>G | 0,812 | 0,666 |
|  | c.594-154G>T | 1,054 | 0,49 |
|  | c.717_718delinsTT | 0,001 | 0,742 |
|  | c.861+101A>G | 1,959 | 0,258 |
|  | c.861+145del | 0,002 | 0,676 |
|  | c.861+145dup | 0,001 | 0,742 |
| *SCNN1D* | c.*204G>A | 0,959 | 0,324 |
|  | c.*254G>A | 0,257 | 0,48 |
|  | c.*259T>C | 1,256 | 0,235 |
|  | c.1031G>C | 0,389 | 0,484 |
|  | c.119+46G>A | 0,969 | 0,51 |
|  | c.1137C>T | 1,336 | 0,513 |
|  | c.1288G>A | 1,054 | 0,49 |
|  | c.1310+11C>T | 1.991 | 0,133 |
|  | c.1310+16G>C | 1,526 | 0,201 |
|  | c.135G>A | 0,001 | 0,742 |
|  | c.1365C>T | 0,969 | 0,51 |
|  | c.1381C>T | 1,054 | 0,49 |
|  | c.1386C>T | 0,803 | 0,319 |
|  | c.1399+20C>T | 1,959 | 0,258 |
|  | c.1399+20C>T | 0,969 | 0,51 |
|  | c.1399+30G>A | 1,959 | 0,258 |
|  | c.1404C>T | 0,969 | 0,51 |
|  | c.1563+27C>T | 0,969 | 0,51 |
|  | c.1630G>C | 1,133 | 0,293 |
|  | c.1663-10C>A | 2,602 | 0,86 |
|  | c.1663-39G>A | 0,757 | 0,27 |
|  | c.1780+39A>C | 0,969 | 0,51 |
|  | c.1781-33G>A | 1,054 | 0,49 |
|  | c.1823G>A | 1,77 | 0,194 |
|  | c.1859+24C>G | 2,014 | 0,365 |
|  | c.1906G>A | 2,129 | 0,237 |
|  | c.1917+28G>A | 0,001 | 0,742 |
|  | c.1918-54A>G | 1.079 | 0,583 |
|  | c.1926T>A | 1,054 | 0,49 |
|  | c.1935G>A | 1,054 | 0,49 |
|  | c.1976+15C>G | 1,34 | 0,199 |
|  | c.1977-22T>C | 0,257 | 0,48 |
|  | c.1977-26G>A | 0,001 | 0,742 |
|  | c.1977-27C>T | 1,054 | 0,49 |
|  | c.2044G>A | 0,169 | 0,52 |
|  | c.2052+19C>T | 0,959 | 0,324 |
|  | c.2070G>C | 2,129 | 0,237 |
|  | c.2109C>T | 1,054 | 0,49 |
|  | c.2176G>A | 1,256 | 0,235 |
|  | c.2216C>G | 1,054 | 0,49 |
|  | c.2308G>A | 0,169 | 0,52 |
|  | c.277+21A>C | 0,292 | 0,41 |
|  | c.277+30G>C | 0,789 | 0,287 |
|  | c.277+68G>A | 1,054 | 0,49 |
|  | c.468G>A | 1,526 | 0,21 |
|  | c.572C>T | 0,969 | 0,51 |
|  | c.6-25G>C | 1,256 | 0,235 |
|  | c.6-54G>T | 0,803 | 0,319 |
|  | c.6-57_6-55del | 0,803 | 0,319 |
|  | c.911+59C>T | 2,034 | 0,168 |
| *TAS1R1* | c.1067C>G | 0,969 | 0,51 |
|  | c.109G>A | 0,969 | 0,51 |
|  | c.1114G>A | 1,243 | 0,537 |
|  | c.1256G>A | 0,969 | 0,51 |
|  | c.1261-14C>T | 1,086 | 0,581 |
|  | c.1261-90C>T | 3,026 | 0,093 |
|  | c.1408A>G | 1,054 | 0,49 |
|  | c.1520G>A | 1,637 | 0,441 |
|  | **c.1594+41G>A** | **4,471** | **0,03** |
|  | c.191+66A>G | 2,102 | 0,35 |
|  | c.191+71C>T | 1,169 | 0,2 |
|  | c.329C>T | 0,389 | 0,484 |
|  | c.498+44G>A | 3,035 | 0,219 |
|  | c.498+84del | 4,177 | 0,124 |
| *TAS1R2* | c.1297G>A | 0,001 | 0,742 |
|  | c,1402T>C | 1,054 | 0,4 |
|  | c.1456A>G | 0,964 | 0,618 |
|  | c.1591+41del | 0,303 | 0,516 |
|  | c.1592+65C>G | 0,969 | 0,51 |
|  | c.1591+67C>G | 0,969 | 0,51 |
|  | c.1719C>T | 0,001 | 0,742 |
|  | c.1720G>A | 0,112 | 0,524 |
|  | c.1784T>C | 1,113 | 0,293 |
|  | c.182+17G>T | 0,303 | 0,516 |
|  | c.183-51C>T | 3.232 | 0,199 |
|  | c.1947C>T | 0,001 | 0,742 |
|  | c.2112C>T | 0,001 | 0,742 |
|  | c.231_233delinsCGT | 0,969 | 0,51 |
|  | c.2319C>T | 4,239 | 0,12 |
|  | c.231T>C | 0,71 | 0,701 |
|  | c.2370C>T | 0,112 | 0,524 |
|  | c.2513G>A | 0,303 | 0,516 |
|  | c.26C>G | 0,903 | 0,637 |
|  | c.484-58G>T | 1,056 | 0,59 |
|  | c.571A>G | 0,668 | 0,716 |
|  | c.789G>A | 1,054 | 0,49 |
|  | c.882T>G | 0,209 | 0,901 |
|  | c.942G>A | 1,054 | 0,49 |
|  | c.949C>G | 0,209 | 0,901 |
| *TAS1R3* | c.*142G>A | 0,002 | 0,676 |
|  | c.*247del | 2,129 | 0,237 |
|  | c.*255C>T | 1,054 | 0,49 |
|  | c.*288G>A | 0,64 | 0,334 |
|  | c.*407C>T | 2,129 | 0,237 |
|  | c.*44C>A | 0,173 | 0,473 |
|  | c.*485A>G | 0,202 | 0,476 |
|  | c.*507C>T | 1,054 | 0,49 |
|  | c.*515T>C | 0,202 | 0,476 |
|  | c.*568C>T | 2,129 | 0,345 |
|  | c.*609C>T | 2,97 | 0,129 |
|  | c.1248C>T | 0,173 | 0,473 |
|  | c.1275+36A>G | 0,969 | 0,51 |
|  | c.1275+9G>A | 2,97 | 0,129 |
|  | c.13_15delinsACA | 0,061 | 0,53 |
|  | c.1362C>T | 0,969 | 0,51 |
|  | c.1363G>A | 0,969 | 0,51 |
|  | c,1601-39T>G | 0,202 | 0,476 |
|  | c.2218C>G | 0,969 | 0,51 |
|  | c.2269= | 0,003 | 0,641 |
|  | c.2407C>T | 0,969 | 0,51 |
|  | c.2511C>G | 1,054 | 0,49 |
|  | c.492+13dup | 0,005 | 0,603 |
|  | c.659G>A | 0,969 | 0,51 |
|  | c.740G>A | 2,129 | 0,237 |
| *TAS2R4* | c.20T>C | 2,075 | 0,354 |
|  | c.286G>C | 2,633 | 0,268 |
|  | c.512G>A | 2,653 | 0,265 |
| *TAS2R10* | c.126G>A | 1,054 | 0,49 |
|  | C.192T>C | 0,01 | 0,742 |
|  | c.231C>T | 1,054 | 0,49 |
|  | c.467C>T | 0,01 | 0,742 |
|  | c.481G>A | 0,969 | 0,51 |
| *TAS2R16* | c.665G>A | 0,956 | 0,62 |
|  | c.837C>T | 1,054 | 0,49 |
|  | c.846G>A | 0,64 | 0,334 |
| *TAS2R38* | c.145G>C | 0,494 | 0,781 |
|  | c.785= | 0,354 | 0,838 |
|  | c.821G>A | 2,129 | 0,237 |
|  | c.886A>G | 0,53 | 0,767 |
|  | c.891G>A | 0,969 | 0,51 |
| *TAS2R43* | c.104G>C | 2,071 | 0,355 |
|  | c.133_150del | 0,97 | 0,616 |
|  | c.198G>C | 0,97 | 0,616 |
|  | c.270G>A | 0,97 | 0,616 |
|  | c.391G>A | 1,466 | 0,48 |
|  | c.460C>G | 0,97 | 0,616 |
|  | c.510T>G | 0,97 | 0,616 |
|  | c.599G>T | 0,97 | 0,616 |
|  | c.63A>G | 0,721 | 0,697 |
|  | c.663C>G | 1,309 | 0,52 |
|  | c.759A>G | 0,97 | 0,616 |
| *TRPV1* | c.*1013C>T | 0,969 | 0,51 |
|  | c.*256T>G | 2,788 | 0,248 |
|  | c.*638del | 0,43 | 0,327 |
|  | c.*708T>G | 0,267 | 0,875 |
|  | c.*1044+103C>T | 0,625 | 0,732 |
|  | c.*1044+74C>T | 0,969 | 0,51 |
|  | c.-121T>C | 0,854 | 0,652 |
|  | c.1224+102G>T | 0,969 | 0,51 |
|  | c.1225-24C>T | 0,168 | 0,92 |
|  | c.1225-29G>A | 3,136 | 0,208 |
|  | c.1383+126A>G | 0,969 | 0,51 |
|  | c.1406C>T | 0,656 | 0,721 |
|  | c.1695T>C | 0,005 | 0,603 |
|  | c.1713+117C>G | 2,464 | 0,292 |
|  | c.-172-14A>G | 0,969 | 0,51 |
|  | c.1753A>G | 2,366 | 0,306 |
|  | c.1780+24G>A | 2,673 | 0,263 |
|  | c.-188_-187del | 1,712 | 0,158 |
|  | c.-188_-187dup | 1,054 | 0,49 |
|  | c.-190_-187del | 2,965 | 0,227 |
|  | c.192_-187del | 1,054 | 0,49 |
|  | c.-196_-187dup | 0,001 | 0,742 |
|  | c.-198_-187dup | 0,112 | 0,524 |
|  | c.2949C>T | 0,081 | 0,527 |
|  | c.2103+35C>A | 0,628 | 0,282 |
|  | c.2103+59_2103+60insAAT | 0,439 | 0,354 |
|  | c.2103+59_2103+60insGAT | 3,355 | 0,055 |
|  | c.2103+62G>T | 0,081 | 0,527 |
|  | c.2104-21G>A | 2,504 | 0,286 |
|  | c.2104-22_2104-21delinsGA | 2,832 | 0,243 |
|  | c.2104-43c>A | 0,173 | 0,473 |
|  | c.2157G>A | 0,173 | 0,472 |
|  | c.2231+11G>A | 0,389 | 0,484 |
|  | c.2231+35G>A | 0,459 | 0,381 |
|  | c.2231+49A>C | 0,389 | 0,484 |
|  | c.2232-85T>C | 2,82 | 0,244 |
|  | c.2238C>T | 0,112 | 0,524 |
|  | c.2347+158C>T | 1,372 | 0,504 |
|  | c.2347+26C>T | 0,969 | 0,51 |
|  | c.254A>G | 0,969 | 0,51 |
|  | c.271C>T | 0,202 | 0,476 |
|  | c.27G>C | 0,969 | 0,51 |
|  | c,284+3A>G | 0,969 | 0,51 |
|  | c.-33-3351C>G | 0,969 | 0,51 |
|  | c.-33-3391C>A | 1,085 | 0,581 |
|  | c.-33-3429T>C | 0,969 | 0,51 |
|  | c.-33-3521G>C | 0,969 | 0,51 |
|  | c.-33-3533C>T | 2,97 | 0,129 |
|  | c.-33-3615C>T | 0,969 | 0,51 |
|  | c.-33-362G>A | 1,284 | 0,526 |
|  | c.-33.390G>A | 1,054 | 0,49 |
|  | c.-33-4516C>T | 0,969 | 0,51 |
|  | c.-33-4598C>A | 1,054 | 0,49 |
|  | c.-33-4634G>C | 4,004 | 0,64 |
|  | c.-33-509_-33-504dup | 4,185 | 0,123 |
|  | c.-33-549G>C | 1,755 | 0,416 |
|  | c.-33-583A>G | 0,969 | 0,51 |
|  | c.501C>T | 0,047 | 0,533 |
|  | c.604+152C>T | 1,054 | 0,49 |
|  | c.604+158C>T | 3,117 | 0,21 |
|  | c.604+36C>T | 1,054 | 0,49 |
|  | c.604+58dup | 0,163 | 0,421 |
|  | c.605-142C>T | 0,969 | 0,51 |
|  | c.605-83C>T | 0,054 | 0,974 |
|  | c.745+13T>C | 1,054 | 0,49 |
|  | c.746-22C>T | 4,352 | 0,54 |
|  | c.945G>C | 0,181 | 0,914 |
|  | c.9A>G | 0,969 | 0,51 |
